# Equal Opportunity and Luck: Empirical Exploration Using the Canadian Longitudinal Study on Aging

**DOI:** 10.1007/s11205-024-03497-3

**Published:** 2024-12-23

**Authors:** Yukiko Asada, Nathan K. Smith, Michel Grignon, Jeremiah Hurley, Susan Kirkland

**Affiliations:** 1 Department of Bioethics, Clinical Center, National Institutes of Health, 10 Center Drive, Bethesda, MD 20892 USA; 2 Department of Economics, Department of Health, Aging and Society, Centre for Health Economics and Policy Analysis (CHEPA), McMaster University, Hamilton, ON L8S4M4 Canada; 3 Department of Economics, Centre for Health Economics and Policy Analysis (CHEPA), McMaster University, Hamilton, ON L8S4M4 Canada; 4 Department of Community Health and Epidemiology, Department of Medicine, Dalhousie University, 5790 University Avenue, Halifax, NS B3H1V7 Canada

**Keywords:** Equality of opportunity, Circumstance, Effort, Luck, Inequality

## Abstract

Equality of opportunity (EOp) is a broad category of egalitarian theories that has attracted considerable attention in recent decades. Empirical implementations of EOp primarily focus on the explained component of inequality, classifying determinants of the outcome (e.g., health) into *effort*—legitimate causes of inequality—and *circumstance*—illegitimate causes of inequality. Largely overlooked is unexplained variation, which in statistical analysis manifests as residuals and is often ignored as a statistical annoyance. The true random component of residuals is now often referred to as *luck*. In this paper, we propose the *playing field* framework that serves as a pragmatic test as to whether residuals signal unfairness in empirical EOp analyses and that enables empirical explorations of roles of luck within the EOp framework. Using a large sample of Canadian older adults, our empirical application of the playing field framework shows that distributions of residuals are not always fair, though there is no consistent pattern of unfairness across age-sex groups. The paper’s three main conclusions are: luck matters; luck should be explicitly incorporated in the EOp framework through the brute luck-effort characterization; and residuals are not just an innocuous statistical annoyance but can represent unfair inequality, and ignoring them can underestimate unfair inequality.

## Introduction

Health inequality is a pressing and persistent concern in many jurisdictions globally. Strict equality in health, however, is rarely identified as the goal, and society typically strives for alleviating unfair health inequality. Egalitarian theories offer diverse accounts as to which health inequality should be considered unfair and why (MacKay & Sreenivasan, 2021). Equality of opportunity (“EOp” henceforth), a broad category of egalitarian theories, has become predominant among egalitarian theories in recent decades (Elford, 2023). EOp states that inequality due to factors beyond individual control is unfair and inequality due to factors within individual control is fair. According to this view, for example, inequality in health resulting from childhood socioeconomic circumstances is unfair, while inequality resulting from a freely chosen commitment to health-enabling behaviours is fair.

In the standard understanding of EOp operationalized for empirical implementation, a person has an opportunity set, exercises freedom, then realizes the outcome (Trannoy, 2016). Early efforts to implement EOp empirically focused on measuring opportunity sets directly. But because opportunity sets are unobservable, measuring them is difficult. As a result, this effort largely died out (Ferreira & Peragine, 2016; Trannoy, 2016). Most empirical EOp work now takes the indirect approach, which builds on the opportunity-freedom-outcome understanding. Two perspectives are possible for the indirect approach. The *ex ante* perspective examines inequality after the opportunity set is revealed but before freedom is exercised. Alternatively, the *ex post* perspective examines inequality after both the opportunity set is revealed and freedom is exercised. In now standard terminology, the revealed opportunity set is referred to as *circumstance* and the exercised freedom as *effort* (Roemer, 1998). EOp is operationalized by either the compensation or reward principle. The compensation principle states that, from the ex ante perspective, inequality between circumstance *types* is to be compensated, whereas from the ex post perspective, inequality within each effort *tranche* (i.e., each effort level) is to be compensated (Ferreira & Peragine, 2016). The reward principle aims to preserve inequality between effort tranches within each circumstance type, and how to meet this aim leads to different versions of the reward principle (Ferreira & Peragine, 2016). EOp does not demand satisfaction of both the compensation and reward principles; in fact, they often conflict, such as, when circumstance and effort correlate (Fleurbaey & Schokkaert, 2011).

Diverse methodological approaches have been developed to implement empirically the compensation and reward principles of EOp. Empirical development has focused primarily on the statistically “explainable” component of inequality (e.g., García-Gómez et al., 2015; Kovacic & Orso, 2022). Researchers have debated extensively which explanatory variables should be classified as circumstance—illegitimate causes of inequality—or effort—legitimate causes of inequality—within the constraints of imperfect real-world data (Jusot & Tubeuf, 2019). They have also examined potential interactions between circumstance and effort (e.g., how family background influences preferences over health behaviours) and their implications for estimates of unfair inequality (Bricard et al., 2013; Jusot et al., 2013). While these issues are important, they are relevant only to the component of inequality that can be explained by circumstance and effort variables. Empirically, however, much variation is left unexplained. Unexplained variation in recent good-quality empirical studies, for example, has accounted for 53–83% of all variation in health (Asada et al., 2018; Jusot et al., 2013), 52–85% in income (Almås et al., 2011; Ferreira & Gignoux, 2008, 2011), and 71–98% in education (Brunori et al., 2010). Unexplained variation in statistical analysis manifests as residuals and is often ignored as a statistical annoyance, largely overlooked in the empirical literature. For example, the increasingly popular empirical implementation of ex ante EOp perspective using the Shapley decomposition method focuses exclusively on the component of inequality explained by circumstance variables, implying residuals are unimportant and uninteresting from the perspective of EOp (Kovacic & Orso, 2022). Whether unexplained variation is classified as fair or unfair, however, drastically changes the estimate of unfair inequality. The estimate of unfair health inequality is 30% greater when classifying unexplained variation as unfair rather than fair (Asada et al., 2015), a much larger impact than the 7% difference observed when the frequently debated ethical status of health behaviours is changed (Asada et al., 2014). How much unfair inequality exists at any given time in society is fundamental information for guiding policy development. The lack of attention to unexplained inequality and its ethical status could misguide social and health policy.

The limited empirical EOp literature has paid attention to unexplained variation through concerns for *randomness* and *residuals*. Trannoy (2016) and Ferreira and Peragine (2016) argue that pervasive randomness in our everyday life cannot be ignored in the EOp framework of circumstance and effort. Fleurbaey and Schokkaert (2011), Roemer and Trannoy (2016), and Jusot and Tubeuf (2019), on the other hand, acknowledge the issue as a problem of residuals or unexplained inequality in econometric models. Residuals include both systematic errors, resulting from omitted variables and model misspecification, and true randomness. True randomness is now often referred to as *luck* (Lefranc et al., 2009; Lefranc & Trannoy, 2017). However, the empirical investigation of luck, and specifically the challenge of identifying the component of unexplained inequality that is luck, is formidable (see, e.g., Bago D’uva et al., 2012; Lefranc et al., 2009).

This paper explores the role of luck in empirical EOp analyses and proposes a pragmatic framework, hereafter referred to as the *playing field*, to guide such analyses. Because a component of residuals is luck, the playing field framework builds on existing typologies of luck and ethical analysis of different types of luck. The playing field assesses covariations between residuals and the type of luck that can be considered unfair and between residuals and the type of luck that can be considered fair. The playing field, thus, serves as a pragmatic test as to whether residuals signal unfairness in empirical EOp analyses. Furthermore, the assessment of covariations between residuals and different types of luck enables empirical explorations of the role of luck within the EOp framework.

The plan of this paper is as follows. As a theoretical foundation for the playing field framework, Sect. 2 synthesizes three well-developed typologies of luck in economics and philosophy and examines which types of luck are considered fair or unfair. Section 3 introduces the playing field framework. Section 4 empirically illustrates this framework using a population-based health dataset and standard parametric econometric methods. Section 5 reports empirical results, showing how residuals covary with a type of luck that can be considered as unfair at least in some population subgroups. Section 6 concludes the paper with three main points: luck plays important roles within the EOp framework; the empirical operationalization of EOp should be reframed from circumstance and effort to brute luck and effort; and the playing field framework suggests residuals are more than an innocuous annoyance in empirical implementations of EOp but can represent unfair inequality.

## Typologies of Luck

We synthesize three prominent typologies of luck and examine the ethical status of luck. The first two—one by Lefranc et al. (2009) and Lefranc and Trannoy (2017) and another by Dworkin (1981a, b)—are familiar in the economics literature. The third, by the philosopher Thomas Nagel, derives from the concept of moral luck (Williams & Nagel, 1976). Despite philosophical inquiry of moral luck over 40 years and its relevance, Nagel’s typology is not widely discussed in the philosophical (but see Lippert-Rasmussen, 2023) and empirical EOp literature. These different typologies share some common conceptions of luck, although different scholars apply different labels to them. All three typologies also align with the opportunity-freedom-outcome understanding central to EOp.

Lefranc et al. (2009) and Lefranc and Trannoy (2017) provide the most comprehensive economics treatment of luck, introducing four types of luck: genetic luck, social circumstance luck, accidental luck, and moral hazard (“LPT typology” henceforth). Luck, they argue, is neither circumstance nor effort, and an examination of types of luck is necessary to determine the ethical status of luck as a cause of inequality. This LPT typology corresponds closely to long-standing philosophical inquiries into luck such as Dworkin’s typology of brute and option luck (1981a, 1981b). Prior to LPT, Dworkin’s typology had arguably been the most frequently relied upon view of randomness in the empirical EOp literature (e.g., Fleurbaey, 2008). The philosopher Bernard Williams offers another influential concept of moral luck (Nelkin, 2021): situations where individuals are morally assessed even though what they are assessed for is beyond their control (Williams & Nagel, 1976). Moral luck threatens the fundamental premise of moral philosophy, since moral assessments of actions taken by individuals apply only to those actions under their control. Responding to Williams, in a third typology, Nagel analyzes moral luck in four ways: resultant, circumstantial, constitutive, and causal luck (Williams & Nagel, 1976).

**Fig. 1:**
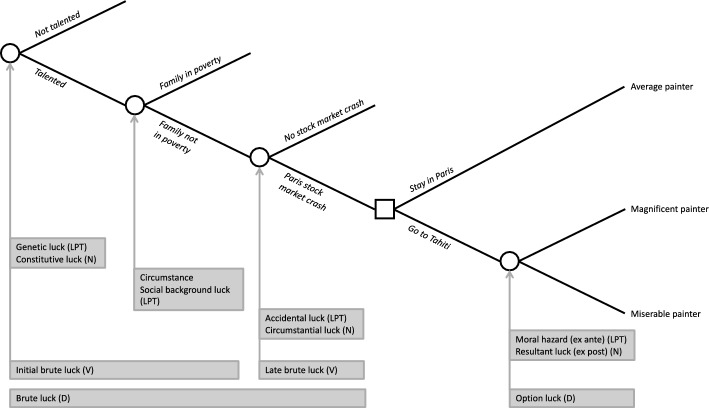
Extended, fictionalized Gauguin and typologies of luck. D: Dworkin; LPT: Lefranc, Pistolesi, Trannoy; N: Nagel; V: Vallentyne

To illustrate these various types of luck, we extend the case of the somewhat fictionalized life of the painter Gauguin originally discussed by Williams and Nagel (1976). All references to Gauguin below are to our scenario. Gauguin’s life consists of four random events (i.e., luck) and one decision point. Figure 1 depicts his life with circles for random events and a square for the decision point. Imagine Gauguin and the associated first random event, whether or not he is talented for painting with a disposition to pursue it. This is what Lefranc et al. (2009) call genetic luck and Nagel constitutive luck: “the kind of person you are, where this is not just a question of what you deliberately do, but of your inclinations, capacities, and temperament” (Williams & Nagel, 1976, p. 140). Assuming he won the genetic talent lottery, Gaugin then experienced the second random event, whether or not the family he was born into was poor. This random event is referred to as circumstance in the EOp framework. Although circumstance is not explicitly called luck, it is a type of luck, a birth lottery related to family and social background, hence referred to as social background luck by LPT (Lefranc & Trannoy, 2017; Lefranc et al., 2009). Genetic luck and social background luck are intertwined; had Gaugin been born into a family in poverty, a talented Gauguin might have ended his life without even noticing or developing his talent. In contrast, if he won both the genetic and birth lottery, a talented Gauguin from a wealthy family continued on in his life, became a successful stockbroker and picked up painting in his free time. He then had the third random event, Paris stock market crash. This is what Lefranc and Trannoy (2017) call accidental luck and Nagel circumstantial luck: “the kind of problems and situations one faces” (Williams & Nagel, 1976, p. 140). Accidental or circumstantial luck may be a health event such as a disease, a period event such as a pandemic, and serendipity such as meeting a good teacher, but for Gauguin, a stock market crash reduced his income but also contributed to his transition to a full-time painter. For Nagel, circumstantial luck includes circumstance in the standard EOp empirical operationalization, and constitutive and circumstantial luck together represents causal luck, “the product of antecedent circumstances outside of the will’s control” (Williams & Nagel, 1976, p. 146).

After some years, Gauguin faced a choice: whether to go to Tahiti or stay in Paris. This choice or decision is referred to as effort in the standard EOp empirical operationalization. This choice is a deliberate gamble for Gauguin—by staying in Paris, he would likely be an average painter; by going to Tahiti, he would either be a magnificent painter or a miserable painter (miserable in the sense that his success as a painter and associated living standard would be much less than in Paris). This outcome is the last type of luck, the one closely connected to the prior decision. Gauguin’s Tahiti decision changed the probabilities of the potential outcomes—average, magnificent, or miserable painter—hence, between the decision and the outcome, there is a luck that is influenced by the decision, and in turn, influences the outcome. From the ex ante perspective—before making the decision and before knowing how the gamble would turn out—Lefranc and Trannoy (2017) call this luck moral hazard, a favourable (unfavourable) shift of the outcome distribution due to the high (low) effort. From the ex post perspective—after making the decision—subsequent randomness—whether Gauguin becomes an average, magnificent, or miserable painter—reflects what Nagel calls resultant luck: “luck, good or bad, in the way things turn out” (Williams & Nagel, 1976, p. 140) and what Dworkin calls option luck: “a matter of how deliberate and calculated gambles turn out—whether someone gains or loses through accepting an isolated risk he or she should have anticipated and might have declined” (Dworkin, 1981b, p. 293). Dworkin contrasts such option luck with brute luck, which he defines as “a matter of how risks fall out that are not in that sense deliberate gambles” (Dworkin, 1981b, p. 293). In our fictionalized case of Gauguin depicted in Fig. 1, brute luck thus includes the three types of luck (genetic/constitutive luck, circumstance, and accidental/circumstantial luck) that happened before the decision point. Vallentyne (2002) further categorized brute luck into initial brute luck—one that happens before the onset of agency (i.e., adulthood)—and late brute luck—one that happens after the onset of agency. Thus, initial brute luck includes genetic/constitutive luck and social background luck, and late brute luck corresponds to accidental/circumstantial luck.

These three typologies of luck offer diverse views as to which types of luck are legitimate or illegitimate causes of inequality. For example, genetic/constitutive luck is the kind of luck that falls under the skin and becomes constituent of an individual. Thus, libertarians who honour individual self-ownership may consider it as a legitimate cause of inequality and argue for preserving its influence on differential outcomes (but then libertarians are unlikely to be interested in egalitarian theories of EOp). Lefranc and Trannoy (2017) discuss a possibility of asymmetrical ethical assessment of good and bad luck: society might wish to respect inequality due to good genetic luck but to compensate for inequality due to bad genetic luck. Vallentyne (2002) considers “initial opportunities for advantage” (initial brute luck) as an illegitimate cause of inequality and argues that any luck before the onset of agency (e.g., adulthood) should be compensated.

Furthermore, the ethical status of option luck has been richly debated. Dworkin argues against compensating bad option luck as it is part of the package for the choice made (1981a, 1981b). Fleurbaey, however, argues that whether or not to compensate bad option luck depends on a person’s degree of risk preference (2008). For Gauguin, Dworkin would not compensate for ending up a miserable painter should Gauguin become one. Fleurbaey would need to know Gauguin’s risk aversion. If Gauguin turned out to be a miserable painter, regretted his decision, and vowed not to take such a gamble again, Fleurbaey would compensate for Gauguin’s loss as a miserable painter. If Gauguin turned out to be a miserable painter, but did not regret his decision, and would take such a gamble again should there be another opportunity, Fleurbaey would not compensate Gauguin. While Dworkin and Fleurbaey consider choice (or effort) and outcomes of the option luck in a binary manner, Lefranc and Trannoy (2017) introduce a distributional consideration for option luck: the benefit (drawback) of high (low) effort includes a favourable (unfavourable) shift of the outcome distribution. According to Lefranc and Trannoy (2017), moral hazard (or option luck) is a legitimate cause of inequality. More precisely, ex ante, Lefranc and Trannoy would respect Gauguin’s decision to go to Tahiti that would shift the outcome distribution favourably to include a possibility of becoming a magnificent painter. Ex post, they would also respect Gauguin’s decision to go to Tahiti, but, when assessing the success of his painting career due to his Tahiti decision, they would account for the option luck that had fallen on him.

Two observations emerge from this synthesis. First, luck plays a critical role in EOp. Indeed, in Roemer’s pragmatic formulation of EOp that became the standard terminology of circumstance and effort, circumstance is originally defined as causes beyond individual control (1993). If taken literally, circumstance in Roemer could have included all types of luck, or at least all forms of brute luck. In Roemer’s operationalization, however, everything that is not captured by observable and measurable circumstance is considered as effort (Lefranc & Trannoy, 2017; Roemer, 1993). LPT moved luck to the forefront of the standard EOp empirical operationalization and advocated that luck be included as a third element to circumstance and effort. However, given that circumstance is one form of luck, we believe that it is more straightforward to frame the EOp framework in terms of luck and effort rather than circumstance, effort, and luck. Therefore, we concur with the literal interpretation of Roemer’s original formulation of circumstance as causes beyond individual control, whether or not it is observable or measurable.

Second, the luck-effort characterization of the EOp framework follows Dworkin’s distinction between brute and option luck: brute luck (i.e., genetic luck, social background luck, and accidental luck) is unfair while option luck is fair. Further, we advocate for the distributional understanding of option luck (i.e., moral hazard), as opposed to the binary understanding of outcomes of option luck. By positing that effort shifts the outcome distribution, the moral hazard view provides clear support for the legitimacy of option luck. In consideration for this dichotomy of the ethical status of brute and option luck, we restate the EOp framework as *brute luck* and effort, with the acknowledgment of effort associated with moral hazard.

This brute luck-effort characterization of the EOp framework, together with the ethical assessment of brute luck as unfair and moral hazard as fair, provides a foundation for the playing field framework introduced in the next section.

## Playing Field Framework

The playing field framework assesses fairness and unfairness of residuals in empirical implementations of EOp through covariations between residuals and brute luck and residuals and effort (including moral hazard). If residuals covary with brute luck, an unfair source of inequality, then, residuals signal unfairness; if residuals covary with effort (and moral hazard), a fair source of inequality, then, residuals do not signal unfairness. The playing field functions both as a pragmatic test as to whether residuals signal unfairness in empirical EOp analyses and as a framework for empirically exploring the roles of luck within the EOp framework.

Below we first define residuals. We then explain how the playing field framework builds on the existing typologies of luck discussed in Sect. 2. Subsequently, we formally define the playing field and how it considers covariations between residuals and brute luck and between residuals and effort as fair or unfair. Introducing the playing field framework, we use a health outcome, but the framework can be applied to other outcomes such as income and education.

Following our formulation of EOp in terms of brute luck and effort, we assume the observed health of an individual ($h$) results from some function ($g$) of luck ($L$) and effort ($E$) of the individual: $h = g(L, E)$. Luck of the individual is determined by some function of brute luck ($B$) and moral hazard ($M$): $L = L(B, M)$. $B$ and $M$ are random variables. B is a random variable that does not depend on any other variable. Moral hazard ($M$), following the characterization by Lefranc and Trannoy (2017), follows the distribution function ($f$) of a random variable parameterized by $E$, where $E$ is not random and is determined by the individual.

In empirical work, the researcher cannot observe all elements of these variables. Hence, brute luck ($B$) comprises observed brute luck ($b$) and unobserved brute luck (${b}^{*}$): $B = B(b, { b}^{*})$, and effort ($E$) comprises observed effort ($e$) and unobserved effort (${e}^{*}$): $E(e, {e}^{*})$. Note how $e$ and ${e}^{*}$ combine to yield $E$ depends on brute luck, hence: $E = E(e, {e}^{*}| b, { b}^{*})$. Following from above, moral hazard follows the distribution function ($f$) of a random variable parameterized by observed effort ($e$) and unobserved effort (${e}^{*}$): $M \leadsto f(e, {e}^{*})$. Empirically, therefore, we predict health based on the health regression: $\widehat{h}=\widehat{g}\left(b,e\right)+\widehat{\varepsilon }\left({ b}^{*}, e, {e}^{*}\right)$. Residuals ($\widehat{\varepsilon })$ capture what we cannot observe, ${b}^{*}$ and ${e}^{*}$, as well as $M$, which is why $\widehat{\varepsilon }$ is a function of $e$.

**Fig. 2:**
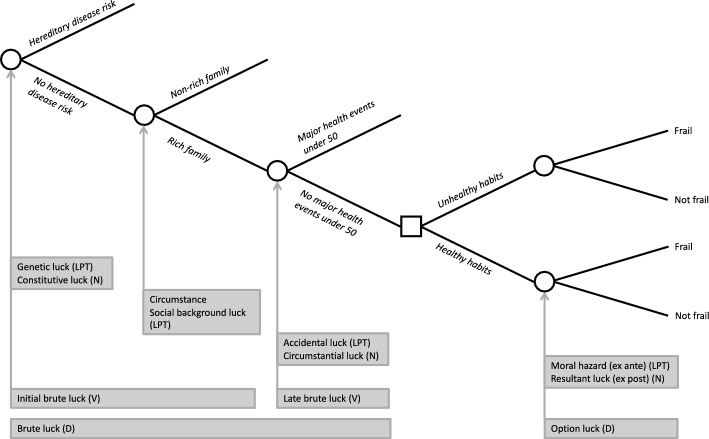
Frailty and typologies of luck. D: Dworkin; LPT: Lefranc, Pistolesi, Trannoy; N: Nagel; V: Vallentyne

To assess fairness and unfairness of the distribution of health residuals, consider a similar schema to the one used for the fictionalized Gauguin case discussed in Sect. 2. Figure 2 depicts the presence of hereditary disease risk (genetic luck), family wealth (social background luck), the presence of major health events under 50 years old (accidental luck), health habits (effort), and option luck associated with health habits to determine the level of frailty of an older person. Recall that brute luck is beyond individual control, thus unfair according to EOp and that option luck, or more precisely, the moral hazard characterization of option luck, is considered fair because it is associated with precedent effort. Because covariation between residuals and brute luck varies by effort, and covariation between residuals and effort varies by brute luck, we ask: do residuals covary with brute luck conditional upon effort, and do residuals covary with effort conditional upon brute luck?

**Fig. 3:**
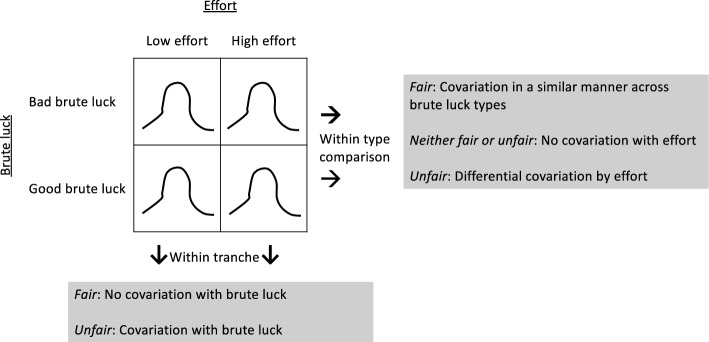
Playing field, within-tranche comparison, and within-type comparison

Formally, to investigate covariations between residuals and brute luck and between residuals and effort, select one particular type of brute luck (i.e., genetic luck, social background luck, or accidental luck) and one particular effort. Consider a matrix for four subgroups of individuals, two brute luck types (bad vs. good) in rows and two effort tranches (low vs. high) in columns (Fig. 3). We call this 2 × 2 matrix the *playing field*. The playing field framework uses that matrix to draw the distribution of residuals for each of the four subgroups of individuals and then measure covariations across tranches or types. Similar to the circumstance-effort matrix comparisons for the standard EOp without consideration for luck (Ferreira & Peragine, 2016), we can make brute luck-effort matrix comparisons. To examine the covariation between residuals and brute luck (i.e., do residuals vary across brute luck conditional upon effort?), we can make *within-tranche* comparisons. To examine the covariation between residuals and effort (i.e., do residuals vary across efforts conditional upon brute luck?), we can make *within-type* comparisons.

When comparing distributions, we posit that greater dispersion of residuals worse than less dispersion of residuals because the former suggests greater uncertainty of the outcome than the latter. Because better brute luck and greater effort are generally associated with better outcomes, we further assume better brute luck and greater effort are associated with less uncertainty of the outcome, that is, less dispersion of residuals.

To assess the fairness and unfairness of the distribution of residuals, within-tranche comparison (column) asks whether the distribution of residuals varies by brute luck conditional upon effort. When the distribution of residuals does not differ by brute luck, that is, there is no covariation between residuals and brute luck, we consider the distribution of residuals to be fair. If we see covariation among either one of the two tranches (low effort, high effort) or both, we conclude there is unfairness in the distribution of residuals. In our health context, brute luck on which we focus is social background luck measured by childhood family wealth (i.e., rich (good luck) vs. non-rich (bad luck)) and the effort on which we focus is health habits (i.e., healthy habits (high effort) vs. unhealthy habits (low effort)). Then, unfairness in the case of covariation might be expressed as follows by the non-rich with unhealthy habits, “I did not practice good health behaviour, but it is unfair I also experience greater uncertainty of the outcome than if I were rich with unhealthy habits, just because of my bad social background luck,” and by the non-rich with healthy habits, “I tried my best to practice good health behaviour. It is unfair, just because of my bad social background luck, that I end up with having greater uncertainty than if I were rich with healthy habits.”

The within-type comparison (row) asks whether the distribution of residuals varies by effort conditional upon brute luck. When the distribution of residuals varies by effort in a similar manner across bad and good brute luck—high effort reduces the degree of uncertainty regardless of brute luck—we consider these distributions of residuals to be fair. When the distribution of residuals varies by effort differently across bad and good brute luck, we consider such differential covariation by brute luck as unfair. The situation when the distribution of residuals does not differ by effort (i.e., no covariation) goes counter to the moral hazard characterization of luck in the sense that good health habits are not fully rewarded in the form of reduced uncertainty of the outcome. We consider no covariation neither fair nor unfair. Assume, as before, that social background luck, measured by family wealth is a type of brute luck and that health habits is a kind of effort. Should residuals covary by effort only among the rich, unfairness might be expressed as follows by the non-rich with healthy habits, “It is unfair that only the rich enjoy the benefit of practicing good health behaviours that reduce uncertainty of the outcome.” Should residuals covary by effort only among the non-rich, the unfairness might be expressed as follows by the non-rich with unhealthy habits, “I accept that I did not practice good health behaviour, but it is unfair that I experience greater uncertainty than the rich with unhealthy habits just because of my bad social background luck.”

In summary, the playing field framework offers a pragmatic test to assess fairness and unfairness of the distribution of residuals. This assessment builds on the well-developed literature on typologies of luck that considers brute luck as an unfair source of inequality and moral hazard as a fair source of inequality. The playing field applies to one particular type of brute luck and one particular kind of effort, thus, it enables an inference as to which type of luck contributes to the unfair distribution of residuals.

## Empirical Strategy

We now explain the data and methods we used to apply the playing field framework to assess empirically unfairness and fairness of health outcomes among a large sample of older adults in Canada. We examine health because brute and option luck likely play a significant role in determining health outcomes, and because effort, in the form of health behaviours, is observable in health. Health behaviours, after adjustment for social background luck, can be considered as factors within individual control (Jusot & Tubeuf, 2019).

*Data:* We used the Canadian Longitudinal Study on Aging (CLSA) (Raina et al., 2009, 2019), a population-based, longitudinal study following approximately 50,000 non-institutionalized Canadians aged 45–85 at the time of recruitment in 2011–2015. For our analysis we used only the baseline, cross-sectional data from the CLSA Comprehensive, a subset of 30,097 participants, who under went in-home face-to-face computer-assisted interviews as well as computer-assisted interviews and clinical and physical assessments at data collection sites. Sampling frames for the CLSA Comprehensive are provincial universal health care registration databases and random digit dialing of landlines. To be included in the sampling frames, potential participants must have resided within a 25 or 50 km radius (depending on the population density) of the 11 data collection sites across Canada. Exclusion criteria included: individuals who spoke neither English nor French; the cognitively impaired at the time of recruitment; residents of the three territories; residents of federal First Nations reserves and other First Nations settlements; and full-time members of the Canadian Armed Forces. After removing 1059 observations due to missing information, the sample size for our analysis was 29,038.

*Health outcome variable:* Our health outcome is frailty, defined as a “decline in functioning across multiple physiological systems, accompanied by an increased vulnerability to stressors” (Hoogendijk et al., 2019) (p. 1365). Frailty is a measure of overall health, well suited to the equal opportunity framework and particularly relevant to studies of older adults. To measure frailty, we constructed the widely-used cumulative deficit based frailty index proposed by Rockwood and Mitnitski (2007). The frailty index counts 30 or more deficits in health, where deficits can be symptoms, signs, diseases, disabilities, or laboratory-assessed abnormalities. We constructed the frailty index using 44 variables available in the CLSA Comprehensive (Appendix 1), ranging from physically assessed performance measures to self-reported chronic conditions. The frailty index is the ratio of the number of deficits present to the total number of deficits considered (e.g., 10/30 = 0.333), where a frailty index of 0.000 indicates the absence of any deficit, and 1.000 indicates the full expression of deficits. Previous studies show that, regardless of the number and specific types of deficits used, the index follows a continuous, gamma distribution; the rate of deficit increase measured by the frailty index is about 0.030 per year; the maximum aggregation of deficits measured by the frailty index is about 0.700 (above which a person dies); and a difference of 0.020 or greater is meaningful as it typically indicates a change in response categories in one of the variables used to construct the frailty index (Fallah et al., 2011; Rockwood & Mitnitski, 2007). To convert the adverse health measure of the frailty index to a positive measure of health, we transformed the frailty index into the “flipped” frailty index (1–frailty index), whereby 0.000 represents the full expression of deficits and 1.000 represents the absence of any deficit. In our analytical sample of 29,038 observations, the flipped frailty index values ranged from 0.353 to 1.000 with the mean value of 0.862 and the standard deviation of 0.089.

*Residuals*: We first estimated the best fit model for the entire analytical sample of 29,038 individuals using unweighted ordinary least squares (OLS) with the flipped frailty index as the dependent variable. We tried log-transformation of the right-skewed frailty index, but it did not yield marked improvement in model fit. Explanatory variables included in the model were: sex, age, age squared, height, height squared, weight, racial/cultural background, immigration status, education, income, household size, home ownership, nutritional risk, marital status, social support, social participation, alcohol consumption, smoking, fruit and vegetable consumption, sleep, physical activity, province, and rurality (see Appendices 2 and 3 for details). These explanatory variables are known to be determinants of health expected to improve the explanatory power of the model (hence, reducing systematic errors in residuals).

Because the purpose of the present analysis was to examine the utility of the playing field framework, improving the explanatory power of the model drove our selection of the explanatory variables. Further, because the present analysis required only the estimation of residuals and did not assess EOp based on the compensation or reward principle, it did not require that we classify these variables to circumstance and effort as in the standard econometric implementation of the EOp framework. Adjusted R-squared for the final model was 0.509.

*Social background luck, household income*: We focused on social background luck as a type of brute luck and used household income as its indicator. Household income in reality would be influenced by both social background luck and effort, and our decision to use it as an indicator of social background luck primarily came from limitations of the dataset, which did not have a variable that clearly and solely captures social background luck. In the playing field analysis, we focused on two extreme ends of the income distribution as they would give the strongest signal for potential covariation between residuals and social background luck. Specifically, we created a binary variable for household income based on the five-category self-reported household income variable available in the CLSA: “Rich” is defined as household income greater than $100,000 versus “Non-rich” defined as household income less than $50,000 or income information missing (who typically are low income). Of the analytical sample of 29,038 observations, 10,089 (34.74%) were classified as rich, 9,341 (32.17%) as non-rich, and 9,608 (33.09%) as neither rich nor non-rich (household income ≥ $50,000 and < $100,000). When applying the playing field framework, we focused on the rich (n = 10,089) and the non-rich (n = 9,608).

*Effort, health behaviours*: As an indicator of effort, we created a binary health-behaviour variable based on smoking, sleeping, and physical activity variables. “Healthy habits” were defined as never smoked, sleeping 6–8 h, and physical activity score in the top 50%; “unhealthy habits” were defined as former or current smoker, sleeping less than 6 h or more than 8 h, and physical activity score in the bottom 50%. Of the analytical sample of 29,038 observations, 6,075 (20.92%) were classified as practicing healthy habits, 1775 (6.11%) as practicing unhealthy habits, and 21,188 (72.97%) as practicing neither healthy nor unhealthy habits. For the playing field analysis, we focused on those with healthy habits (n = 6075) and those with unhealthy habits (n = 1775).

*Playing fields*: Following the common epidemiological stratifications, we defined the playing field by sex (male vs. female) and age groups (45–54, 55–64, 65–74, and 75–84 years). This approach assumes that it is reasonable to compare distributions of residuals by social background luck, measured by income, and effort, measured by health behaviour, among persons in the same age-sex group. We thus had eight playing fields within each of which we assessed fairness and unfairness of distributions of residuals by social background luck and effort. In addition to the overall model described above, we ran stratified models by age, obtained predicted values, and calculated residuals for individuals in each age-sex category to which we applied the playing field framework.

*Analysis*: The analysis focused on the 5393 participants who were classified as either rich or non-rich and as practicing either healthy habits or unhealthy habits. In each of the eight age-sex playing fields we applied the within-tranche and the within-type comparisons, as discussed in Sect. 3. To determine if two distributions were different, we used three methods. First, we visually inspected kernel density plots of distributions of residuals. Second, we compared the standard deviations of the distributions. Third, we used the Kolmogorov–Smirnov test of equality of distributions, which is based on cumulative distribution functions, has been used in EOp empirical work on health (Bricard et al., 2020; Rosa Dias, 2009; Trannoy et al., 2009), and is more sensitive than the median-based Kruskal–Wallis test used by Bago d’Uva et al. (2012). Furthermore, given the large number of comparisons in our analysis—two comparisons within tranche and two comparisons within type for each of eight playing fields, resulting in 32 comparisons in total—we applied the Bonferroni correction to reduce false-positive results. Because the primary purpose of our analysis is to illustrate our methodological approach and not to draw inferential conclusions, our analysis was not weighted by survey weights, and variance estimates did not account for the complex survey design of the CLSA.

## Empirical Results

Table 1 shows results of the within-tranche and the within-type comparisons of distributions of residuals in eight playing fields. For each of the four subgroups in each of the eight playing fields (e.g., the non-rich with unhealthy habits among 45–54 years old males), the top row number (e.g., 45) is the sample size and the bottom row number (e.g., 0.081) is the standard deviation of the distribution of residuals. The row *p*-values at the bottom of each playing field (e.g., 0.637 and 0.268) are for the Kolmogorov–Smirnov test for the within-tranche comparison of distributions of residuals (one for the healthy-habit and one for the unhealthy-habit tranche). When one of these row *p*-values is statistically significant (i.e., *p* < 0.0125), we consider the distribution of residuals by effort to be unfair.

**Table 1:**
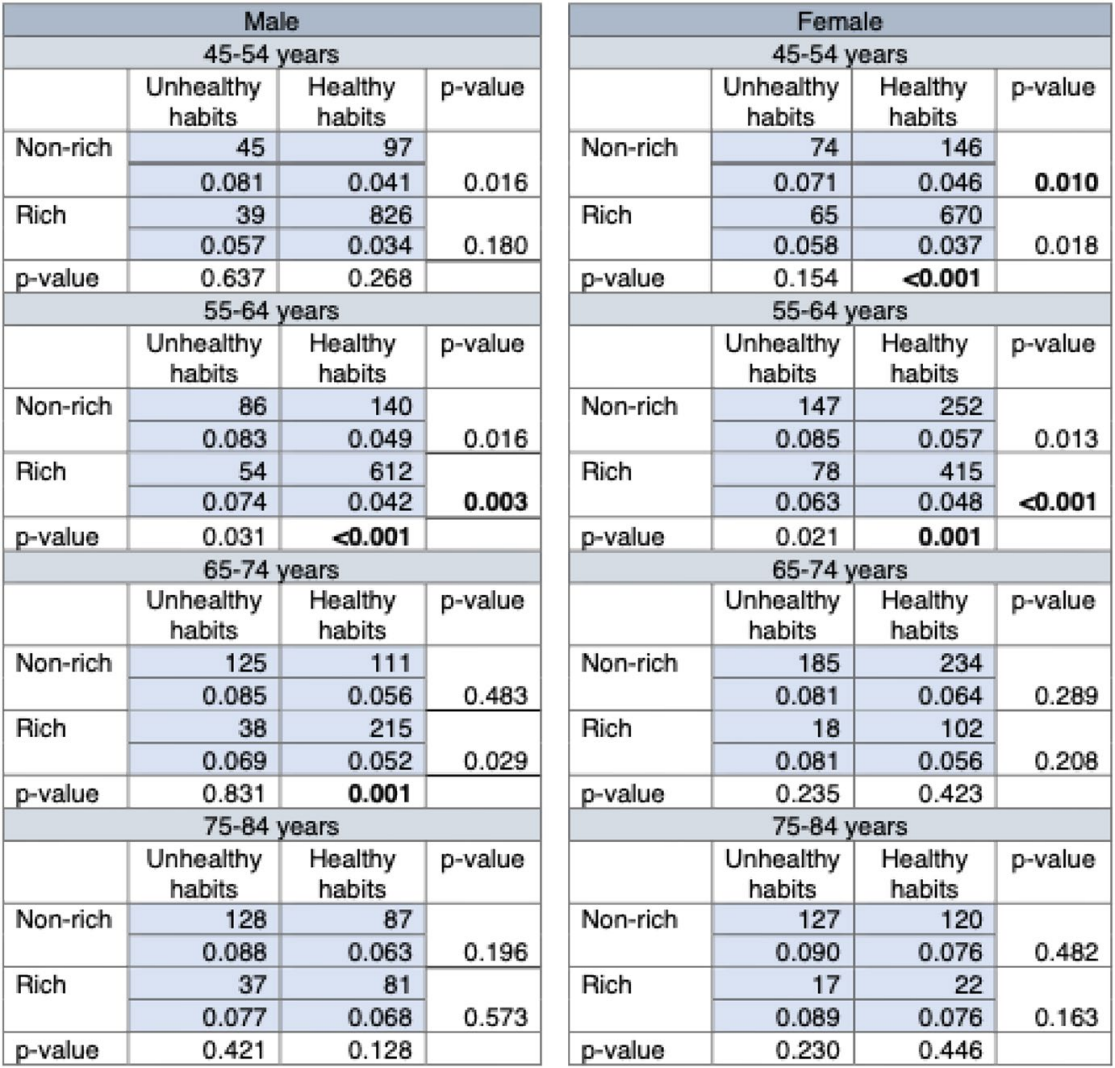
Sample size, standard deviation, and *p*-value for the Kolmogorov–Smirnov test in playing fields

For each of the four subgroups in each of the eight playing fields (e.g., the non-rich with unhealthy habits among 45–54 years old males), the top row number (e.g., 45) is the sample size and the bottom row number (e.g., 0.081) is the standard deviation of the distribution of residuals. The row *p*-values at the bottom of each playing field (e.g., 0.637 and 0.268) are for the Kolmogorov–Smirnov test for the within-tranche comparison of distributions of residuals (one for the healthy-habit and one for the unhealthy-habit tranche). The column *p*-values at the furthest column of each playing field (e.g., 0.016 and 0.180) are for the Kolmogorov–Smirnov test for the within-type comparison of distributions of residuals (one for the rich and one for the non-rich). We consider *p* < 0.0125 as statistically significant, written in bold

The column *p*-values at the furthest column of each playing field (e.g., 0.016 and 0.180) are for the Kolmogorov–Smirnov test for the within-type comparison of distributions of residuals (one for the rich and one for the non-rich). When one of these column *p*-values is statistically significant but another is not (i.e., residuals covary with effort differentially by social background luck), or when both of the column *p*-values are statistically not significant (i.e., “effort does not pay” in the sense that it did not alter the distribution of luck), we consider the distribution of residuals by social background luck to be unfair. Applying the Bonferroni correction, we consider *p* < 0.0125 as statistically significant; significant results are written in bold.

The prevalence of healthy and unhealthy habits across age-sex groups in Table 1 exhibits a noteworthy age-related pattern. Whereas healthy habits are much more prevalent than unhealthy habits among 45–54 and 55–64 years old males and females, unhealthy habits are more prevalent than or almost equally prevalent to healthy habits among 65–74 and 75–84 years old males and females, especially among the non-rich. The reasons for this are not known, but it may be that: (1) older people think investment in healthy habits is of less value than younger people, especially among the non-rich; and/or (2) older people are less able to practice healthy habits due to declining health.

**Fig. 4:**
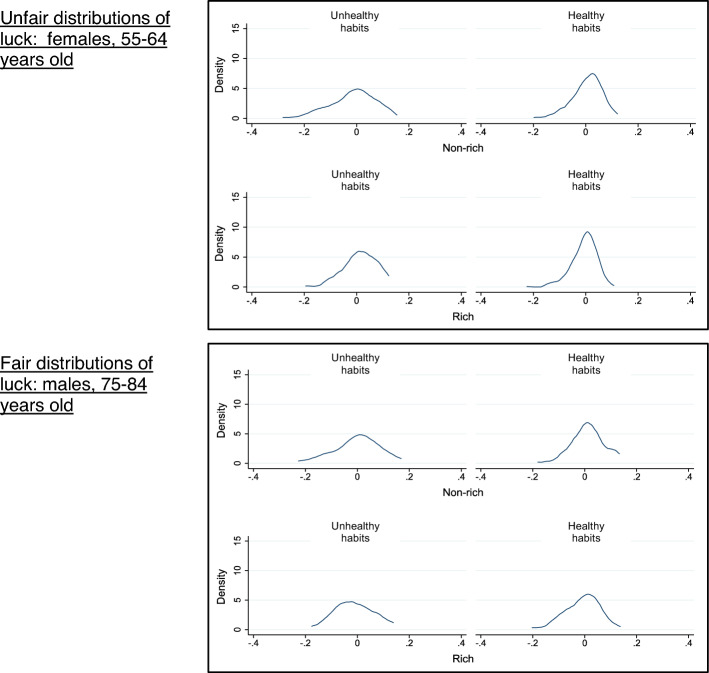
Examples of distributions of luck by circumstance and effort, 55–64 years female and 75–84 years male. Note: Distributions for 55–64 years old females show covariation of residuals both with social background luck and effort, and distributions for 75–84 years old males show no covariation between residuals and social background luck and between residuals and effort. The statistical assessment of these distributions is in Table 1

The standard deviation of each subgroup in Table 1 largely confirms our assumption that better social background luck and greater effort are associated with narrower distributions of residuals. Within each playing field, the smallest standard deviation is always for the rich with healthy habits, whereas the largest standard deviation is often for the non-rich with unhealthy habits. The Kolmogorov–Smirnov test results in Table 1 show that residuals covary with social background luck among males with healthy habits in the 55–64 and 65–74 age groups and among females with healthy habits in the 45–54 and 55–64 age groups (within-tranche comparison), thus, residuals are unfairly distributed among them. The top graph in Fig. 4 is an example of such unfair distribution of residuals. Among 55–64 age group females with healthy habits, the distribution of residuals is narrower among the rich than among the non-rich (within-tranche comparison).

Residuals covary with effort among the rich but not among the non-rich both for males and females in the 55–64 age group, and residuals covary with effort among the non-rich but not among the rich for females in the 45–54 age group (within-social background type comparison), thus, residuals are unfairly distributed among these groups. The same top graph in Fig. 4, as before, is also an example of such unfair distribution of residuals. Among rich 55–64 age group females, the distribution of residuals is narrower among those with healthy habits than among those with unhealthy habits, but such covariation between residuals and effort is not observed among the non-rich. In addition, among males in the 45–54, 65–74, and 75–84 age groups and among females in the 65–74 and 75–84 age groups, residuals do not covary with effort, thus, residuals are neither fairly nor unfairly distributed among these groups. The bottom graph in Fig. 4 is an example of such a distribution of residuals that is neither fair nor unfair. Among rich and non-rich 75–84 age group males, the distribution of residuals is similar among those with healthy habits and among those with unhealthy habits.

Taken together, as further summarized in Table 2, males and females in the 55–64 age group and females in the 45–54 age group have unfair distributions of residuals in terms of covariations with both social background luck and effort, and males in the 65–74 age group have unfair distributions of residuals in terms of covariation with social background luck.

**Table 2:** Summary of unfair distributions of residuals

Age group	Male		Female	
	Covariation with social background luck	Covariation with effort	Covariation with social background luck	Covariation with effort
45–54	Fair	Neither fair or unfair	Unfair	Unfair
55–64	Unfair	Unfair	Unfair	Unfair
65–74	Unfair	Neither fair or unfair	Fair	Neither fair or unfair
75–84	Fair	Neither fair or unfair	Fair	Neither fair or unfair

## Discussion

Acknowledging the importance of luck in the EOp framework but faced with the challenges of disentangling luck from residuals in the empirical EOp analyses, we sought to develop a pragmatic framework to assess the ethical status of residuals. Drawing inspiration from the literature on luck in philosophy and economics, we reframed the standard circumstance-effort EOp characterization by Roemer as the brute luck-effort characterization with the acknowledgment of effort associated with moral hazard. We proposed the playing field framework that assesses the unfairness and fairness of residuals in terms of covariation between residuals and brute luck (unfair) and between residuals and effort, which is associated with moral hazard (fair). Using a sample of Canadian older adults, we empirically applied the playing field framework. Both covariations between residuals and brute luck and between residuals and effort suggest that distributions of residuals are not always fair, though there is no consistent pattern of unfairness across age-sex groups.

The playing field framework is primarily a pragmatic test for the ethical status of residuals. Yet, because it makes the ethical assessment based on the typologies of luck and the ethical analysis of different types of luck, it can also serve as a framework that assists empirical exploration of luck. Empirical analysis of luck within the EOp framework to date is limited because it is challenging to identify the component of residuals that is luck and distinguish different types of luck, as in the case for two pioneer studies (Lefranc et al., 2009 and Bago d’Uva et al., 2012). Instead of attempting to estimate luck directly, or assuming that residuals are a reasonable approximation of luck, the playing field explores luck indirectly through covariations between residuals and observable brute luck and between residuals and observable effort. In our empirical implementation, we chose social background luck as an example of brute luck. Should data be available, it would be possible to apply the playing field analysis to other types of brute luck, for example, genetic luck, measured by the existence of hereditary disease in the family or the age of death of parents, and accidental luck, measured by the existence of major health events under the age of 50.

As a proof-of-concept of the playing field framework, our empirical analysis did not extend to the standard EOp analysis. In future work, as a pragmatic test for the ethical status of residuals, the playing field can supplement empirical EOp analyses using the standard parametric econometric methods. The playing field forces analysts to pay attention to residuals and can indicate whether disregarding them in the analysis may underestimate unfairness. Using the playing field to strengthen empirical EOp analyses, future work needs to select carefully variables indicating brute luck. In this study, among different types of brute luck, we focused on social background luck and used household income as an indicator. Household income admittedly is not the best indicator for social background luck as it represents both social background luck and effort. Our choice was limited with the availability of variables in the dataset we used. Variables that capture parental information and/or adverse childhood experience (Chen et al., 2022; Kovacic & Orso, 2022) are a promising candidate to capture social background luck.

This study demonstrated the utility of the playing field framework, but its implementation posed important questions such as the criteria used to compare residual distributions, the use of the age-sex group within which to compare residual distributions, and the size of data to implement the playing field. These issues need to be examined more thoroughly in future work. The question of the goodness of the shape of distributions of residuals deserves further discussion, for example, whether, and if so, how, the skewness of the distribution (as indicated by residual distributions in Fig. 4) should play a role in our assessment of goodness of the shape of distributions, and why we might consider a certain shape of distribution of residuals as worse than others. These questions connect to the discussion regarding asymmetric compensation of genetic luck in Lefranc and Trannoy (2017): we may wish to compensate bad luck, but we may wish to allow people enjoy good luck. Methodologically, there is no single, clearly preferred method for testing equivalence of two distributions. We opted for the commonly used Kolmogorov–Smirnov test, but future work should explore alternative methods.

Furthermore, it is important to consider how to define the population groups for whom it is appropriate to compare distributions of residuals. Our age-sex definition of the playing field has face validity as it follows the common epidemiological stratifications with the understanding that health differences by age and between sexes are to be expected and are acceptable. In addition, if we assume unfair health may manifest differently between males and females, sex stratification of the playing field analyses is appropriate. Implicit in this view is the understanding of age and sex as biological determinants of health, hence, differences by age and sex are uninteresting in the *ethical* examination of the distribution of health. Age and sex, however, are both biological and social determinants of health, supported by a simple observation of a wide range of health outcomes among people of the same age and a now common distinction between sex (biological determinant) and gender (social determinant). We acknowledge thoughtful discussion should be made to define an appropriate playing field but defer careful discussion on these issues to future work. Finally, it would be useful to determine a reasonable sample size for the playing field analysis. It is desirable to have a large sample size for the stratified distributional analysis the playing field analysis calls for, and it is also preferable to contrast four groups in the 2 × 2 playing fields, as we opted to focus on two extremes of social background luck and effort in our analysis. Taken together, the playing field analysis demands large data sets, but what the minimum sample size is for a reasonable analysis requires future exploration. To sum, the lack of consistent pattern of unfair distributions of residuals across age-sex groups observed in this study calls for future studies applying the playing field framework to richer data that enable the use of more sophisticated methods to distinguish fair and unfair determinants. Only then can we confidently assess potential age-sex patters of unfair distributions of residuals. This study can be considered as a first step aimed at illustrating the potential of the approach.

We conclude this paper with three key points that emerged from the development and empirical application of the playing field. First, luck matters. The existing typologies of luck offer further vocabulary to enrich our understanding and empirical exploration of EOp. Second, the operationalization of the EOp framework can benefit from explicitly incorporating luck. For the development of the playing field, we classified circumstance as a type of luck and reframed the standard circumstance-effort operationalization of EOp (Roemer, 1998) as brute luck-effort with the acknowledgment that effort is associated with option luck, which is characterized as moral hazard (Lefranc & Trannoy, 2017). Finally, the playing field framework is useful, and it showed that residuals are unlikely just an innocuous statistical annoyance but can represent unfair inequality. Given that residuals are often more than 50% of observed inequality, ignoring them can result in vast underestimation of unfair inequality. Our results suggest such underestimation is possible, and the playing field can be used as a method to identify and mitigate potential underestimation.

## Appendix 1 Construction of the Frailty Index

- In the literature, criteria discussed in Searle et al (2008) appear to be most common to select variables to construct the frailty index:

1. Variables must be deficits associated with health status.
2. The deficit must increase in prevalence along with age.
3. The deficit shouldn’t be overly common in late age.
4. The selected deficits must cover a range of different body systems.
5. If the frailty index was used to follow up the same person, the composition of the frailty index must be exactly the same across the follow-ups.

- Blodgett (2014) operationalizes the criteria above and add a criterion regarding missing (#2 below). We used these criteria:

1. Variables must be deficits associated with health and age conceptually.
2. The deficit should not have too many missing data (missing in more than 5% of the study population).
3. The deficit should be common (present in at least 1% of the study population).
4. The deficit shouldn’t be overly common in late age (Blodgett: deficit was present in 80% or more of individuals by age of 80; for our analysis, we modified to 80% or more of individuals by age of 70).
5. The deficit must increase in prevalence along with age.
6. The selected deficits must cover a broad range of body systems.

- The flowchart next page describes the variable selection applying these six criteria. On subsequent pages, we list 44 variables selected to construct the frailty index.

Summary of the Variable Selection

Variable Selected to Construct the Frailty Index

**Table:**

Variable	CLSA variable source*	N (%) missing	Prevalence (%) overall	Prevalence (%) at age 70
Function (9)				
Vision (self-rated)	vis_sght_com	19 (0.06%)	7.67	6.77
Hearing: clinically measured (right)	hrg_right_1k_com	1253 (4.16%)	13.70	8.20
Hearing: clinically measured (left)	hrg_left_1k_com	844 (2.80%)	13.12	7.65
Hearing (self-rated)	hrg_hrg_com	28 (0.09%)	11.42	9.63
OARS scale: Able to walk	adl_ablwk_com	3 (0.01%)	2.08	1.43
Slow walking speed (4 Meter walk)	wlk_time_com	372 (1.24%)	20.13	14.63
Timed get up and go (TUG)	tug_time_com	411 (1.37%)	5.02	2.35
5 Repetition chair rise	cr_time_com	1315 (4.37%)	4.98	3.59
Sense of balance (self-rated)	pkd_bal_com	169 (0.56%)	13.07	9.12
Cognition Measures (4)				
Rey Auditory Verbal Learning—Immediate (First Recall)	cog_reyi_score_com	1020 (3.39%)	9.78	6.13
Rey Auditory Verbal Learning—Delayed (Delayed Recall)	cog_reyii_score_com	1051 (3.49%)	40.42	33.15
Mental Alternation Test (MAT)	cog_mat_score_com	1486 (4.94%)	5.46	3.98
Animal Fluency Test (AFT)	cog_aft_score_1_com	731 (2.43%	6.97	4.47
Activity Of Daily Living (1)				
OARS scale: Trouble to get in time to bathroom	adl_bath_com	34 (0.11%)	13.06	10.42
Instrumental Activity of Daily Living (IADL) (1)				
OARS scale: Able to do housework	ial_ablwrk_com	8 (0.03%)	4.52	2.88
Oral health (2)				
Has one or more or original teeth	orh_teeth_mcq	1310 (4.35%)	6.55	4.03
Wears dentures or false teeth	orh_dent_mcq	1308 (4.35%)	25.27	18.93
Other (4)				
Broken bone from fall or injury (during adult life)	ost_bone_com	131 (0.44%)	14.96	13.45
Coughed most days—last 12 months	cao_cofpy_com	125 (0.42%)	15.81	14.85
Have a prosthetic limbs or joint	icq_proslim_com	3 (0.01%)	5.94	3.58
Cough, choke when swallowing food	nur_swllfd_mcq	1343 (4.46%)	39.33	38.02
Chronic conditions (19)				
Other type of arthritis	ccc_artot_com	401 (1.33%)	10.41	9.83
Osteoarthritis in one or both hands	ccc_oahand_com	324 (1.08%)	12.95	10.72
Osteoarthritis in the hip	ccc_oahip_com	353 (1.17%)	8.40	6.68
Osteoarthritis in the knee	ccc_oaknee_com	387 (1.29%)	15.14	13.18
Osteoporosis	ccc_ostpo_com	290 (0.96%)	9.02	6.77
High blood pressure/hypertension	ccc_hbp_com	179 (0.59%)	37.10	31.64
Emphysema, chronic bronchitis, COPD, or chronic changes in lungs due to smoking	ccc_copd_com	185 (0.61%)	5.77	4.85
Heart disease (including congestive heart failure, or CHF)	ccc_heart_com	183 (0.61%)	11.71	8.03
Heart attack or myocardial infarction	ccc_ami_com	149 (0.50%)	4.88	3.36
Angina	ccc_angi_com	170 (0.56%)	4.42	2.90
Peripheral vascular disease or poor circulation in limbs	ccc_pvd_com	173 (0.57%)	5.50	4.24
Cancer	ccc_canc_com	93 (0.31%)	15.45	11.64
Pain or paralysis in hands or wrists	icq_painhnd_com	1 (0.00%)	19.62	17.75
Macular degeneration	ccc_macdeg_com	214 (0.71%)	4.28	2.29
UNDER-active thyroid gland	ccc_uthyr_com	394 (1.31%)	13.34	11.69
Ever suffer from cataracts	icq_catrct_com	621 (2.06%)	29.23	16.32
Ever had glaucoma	icq_glauc_com	164 (0.54%)	5.09	3.20
Experienced an effect from mini-stroke or transient ischemic attack (TIA)	ccc_tia_com	242 (0.80%)	3.23	1.91
Urinary incontinence	ccc_uriinc_com	103 (0.34%)	8.39	6.44
Cognitive condition (1)				
CES-D 10 scale: frequency feel lonely	dep_lonly_com	121 (0.40%)	2.13	2.01
Lab test (3)				
Average systolic blood pressure	bp_systolic_all_avg_com	286 (0.95%)	51.09	46.26
Average pulse rate	bp_pulse_all_avg_com	289 (0.96%)	16.79	15.28
Electrocardiography (ECG) Diagnosis Result	ecg_result_com	601 (2.00%)	40.98	36.11

*Canadian longitudinal study on aging datasets < https://datapreview.clsa-elcv.ca/datasets >

## Appendix 2 Sample Characteristics by Variables Included in the Regression Model

**Table:**

Variable	Note	N	% or mean	Flipped frailty index
Sex				
Male		14,277	49.17	0.866
Female		14,761	50.83	0.858
Age		29,038	62.86	0.862
Height (cm)		29,038	168.37	0.862
Weight (kg)		29,038	79.81	0.862
Racial/cultural background				
Black		211	0.73	0.851
East/Southeast Asian		351	1.21	0.880
Indigenous		92	0.32	0.839
Latino		97	0.33	0.881
Middle Eastern		118	0.41	0.881
South Asian		258	0.89	0.853
White		27,403	94.37	0.862
Other		81	0.28	0.851
Multi-racial		427	1.47	0.868
Immigration status				
Non-immigrant	Length of time since immigrated to Canada	23,819	82.03	0.863
Less than 15 years ago		479	1.65	0.906
15–29 years ago		665	2.29	0.887
30–44 years ago		1484	5.11	0.868
45 + years ago		2591	8.92	0.837
Education				
Less than high school		1574	5.42	0.782
High school graduation		2747	9.46	0.842
Some post-sec/trade		5378	18.52	0.844
College/university		6192	21.32	0.863
Bachelor’s degree		6853	23.60	0.883
Degree > bachelor’s		6294	21.68	0.883
Household income				
< $20,000		1451	5.00	0.795
$20,000 to < $50,000		6091	20.98	0.821
$50,000 to < $100,000		9608	33.09	0.863
$100,000 to < $150,000		5390	18.56	0.893
$150,000 +		4699	16.18	0.910
Missing		1799	6.20	0.833
Household size				
1	Number of people in household	6493	22.36	0.824
2		14,178	48.83	0.857
3		4041	13.92	0.891
4		2981	10.27	0.912
5		941	3.24	0.911
6 +		404	1.39	0.895
Home ownership				
Rent		4328	14.90	0.820
Own		24,710	85.10	0.870
Nutritional risk				
Not high risk	Measured by AB SCREEN (TM) II (Abbreviated Seniors in the Community Risk Evaluation for Eating and Nutrition II)	17,811	61.34	0.877
High risk		9672	33.31	0.839
Missing		1555	5.36	0.833
Marital status				
Married/common-law		20,056	69.07	0.874
Widowed/divorced/separated		6448	22.21	0.824
Single, never married		2534	8.73	0.864
Social support				
Low availability	Measured by the 19-item, self-administered Medical Outcomes Study (MOS) Social Support Survey	12,244	42.17	0.845
High availability		16,794	57.83	0.875
Social participation				
At least once a year or none	Frequency of community-related activity participation	451	1.55	0.813
At least once a month		3638	12.53	0.858
At least once a week		19,868	68.42	0.863
At least once a day		5081	17.50	0.866
Alcohol				
Never drink		685	2.36	0.839
Former drinker		3257	11.22	0.832
Occasional drinker		8321	28.66	0.851
Regular drinker		12,383	42.64	0.870
Regular heavy drinker		4392	15.13	0.884
Smoking				
Never smoked		13,861	47.73	0.874
Former smoker		12,708	43.76	0.851
Current smoker		2469	8.50	0.852
Daily fruit/vegetable				
< 2 servings		2201	7.58	0.849
2 to < 4 servings		11,288	38.87	0.861
4 to < 6 servings		10,537	36.29	0.863
6 to < 8 servings		3729	12.84	0.868
> 8 servings		1283	4.42	0.877
Sleep				
< 6 h	Based on the International Classification of Sleep Disorders	3797	13.08	0.839
$$\ge$$ 6 and < 9 h		23,627	81.37	0.868
$$\ge$$ 9 h		1614	5.56	0.832
Physical activity				
First quarter	Measured by the Physical Activity Scale for the Elderly (PASE) scores	7368	25.37	0.818
Second quarter		7235	24.92	0.854
Third quarter		7180	24.73	0.877
Fourth quarter		7255	24.98	0.900
Province				
Newfoundland and Labrador		2156	7.42	0.859
Nova Scotia		2956	10.18	0.861
Quebec		5821	20.05	0.856
Ontario		6228	21.45	0.862
Manitoba		2995	10.31	0.858
Alberta		2830	9.75	0.864
British Columbia		6052	20.84	0.871
Rurality				
Urban		26,673	91.86	0.861
Rural		2365	8.14	0.872

Canadian longitudinal study on aging comprehensive, Baseline data, Unweighted

## Appendix 3 Regression model based on which luck was estimated

**Table:**

Variable	Coefficient	SE	t	P >| t |	99% CI low	99% CI high
Sex						
Male (ref)						
Female	− 0.004	0.001	− 3.570	0.000	− 0.007	− 0.001
Age	0.004	0.000	8.010	0.000	0.003	0.005
Age squared	0.000	0.000	− 18.230	0.000	0.000	0.000
Height (cm)	0.004	0.001	3.510	0.000	0.001	0.006
Height squared	0.000	0.000	− 2.550	0.011	0.000	0.000
Weight (kg)	− 0.001	0.000	− 37.680	0.000	− 0.001	− 0.001
Racial/cultural background						
Black	− 0.008	0.004	− 1.720	0.086	− 0.019	0.004
East/Southeast Asian	− 0.007	0.004	− 1.980	0.048	− 0.016	0.002
Indigenous	− 0.009	0.007	− 1.300	0.194	− 0.025	0.008
Latino	0.001	0.007	0.160	0.876	− 0.016	0.018
Middle Eastern	0.000	0.006	− 0.010	0.990	− 0.016	0.015
South Asian	− 0.017	0.004	− 4.050	0.000	− 0.027	− 0.006
White (ref)						
Other	− 0.008	0.007	− 1.190	0.235	− 0.026	0.010
Multi-racial	− 0.005	0.003	− 1.590	0.111	− 0.013	0.003
Immigration status						
Non-immigrant (ref)						
Less than 15 years ago	0.004	0.003	1.380	0.167	− 0.004	0.012
15 to 29 years ago	− 0.002	0.003	− 0.660	0.506	− 0.009	0.005
30 to 44 years ago	0.004	0.002	2.100	0.036	− 0.001	0.008
45 + years ago	0.003	0.001	2.340	0.020	0.000	0.007
Education						
Less than high school (ref)						
High school graduation	0.019	0.002	9.420	0.000	0.014	0.024
Some post-sec/trade	0.015	0.002	8.180	0.000	0.010	0.020
College/university	0.022	0.002	11.790	0.000	0.017	0.026
Bachelor’s degree	0.027	0.002	14.220	0.000	0.022	0.031
Degree > bachelor’s	0.027	0.002	14.070	0.000	0.022	0.032
Household income						
< $20,000 (ref)						
$20,000 to < $50,000	0.018	0.002	9.460	0.000	0.013	0.023
$50,000 to < $100,000	0.028	0.002	14.160	0.000	0.023	0.033
$100,000 to < $150,000	0.032	0.002	14.820	0.000	0.026	0.038
$150,000 +	0.033	0.002	14.690	0.000	0.027	0.039
Missing	0.022	0.002	9.400	0.000	0.016	0.028
Household size						
1 (ref)						
2	− 0.002	0.001	− 1.080	0.281	− 0.005	0.002
3	− 0.001	0.002	− 0.640	0.522	− 0.005	0.003
4	− 0.002	0.002	− 0.960	0.336	− 0.007	0.003
5	− 0.004	0.003	− 1.740	0.083	− 0.011	0.002
6 +	− 0.005	0.003	− 1.370	0.170	− 0.014	0.004
Home ownership						
Rent	− 0.010	0.001	− 8.890	0.000	− 0.014	− 0.007
Own (ref)						
Nutritional risk						
Not high risk (ref)						
High risk	− 0.019	0.001	− 22.050	0.000	− 0.021	− 0.017
Missing	− 0.006	0.002	− 3.110	0.002	− 0.010	− 0.001
Marital status						
Married/common-law (ref)						
Widowed/divorced/separated	0.000	0.001	0.230	0.817	− 0.003	0.004
Single, never married	0.006	0.002	3.480	0.001	0.002	0.011
Social support						
Low availability (ref)						
High availability	0.009	0.001	10.910	0.000	0.007	0.011
Social participation						
At least once a year or none (ref)						
At least once a month	0.014	0.003	4.580	0.000	0.006	0.022
At least once a week	0.017	0.003	5.580	0.000	0.009	0.025
At least once a day	0.018	0.003	5.740	0.000	0.010	0.026
Alcohol						
Never drink (ref)						
Former drinker	− 0.005	0.003	− 1.770	0.076	− 0.012	0.002
Occasional drinker	0.003	0.003	1.050	0.292	− 0.004	0.009
Regular drinker	0.009	0.003	3.580	0.000	0.003	0.016
Regular heavy drinker	0.008	0.003	3.010	0.003	0.001	0.015
Smoking						
Never smoked (ref)						
Former smoker	− 0.008	0.001	− 10.060	0.000	− 0.010	− 0.006
Current smoker	− 0.019	0.001	− 13.300	0.000	− 0.023	− 0.016
Daily fruit/vegetable						
< 2 servings (ref)						
2 to < 4 servings	− 0.002	0.001	− 1.030	0.302	− 0.005	0.002
4 to < 6 servings	− 0.003	0.002	− 1.980	0.048	− 0.007	0.001
6 to < 8 servings	− 0.005	0.002	− 2.660	0.008	− 0.009	0.000
> 8 servings	− 0.004	0.002	− 1.880	0.060	− 0.010	0.002
Sleep						
< 6 h	− 0.014	0.001	− 12.910	0.000	− 0.017	− 0.011
$$\ge$$ 6 and < 9 h (ref)						
$$\ge$$ 9 h	− 0.008	0.002	− 4.740	0.000	− 0.012	− 0.004
Physical activity						
First quarter (ref)						
Second quarter	0.016	0.001	14.390	0.000	0.013	0.018
Third quarter	0.022	0.001	19.430	0.000	0.019	0.025
Fourth quarter	0.021	0.001	17.440	0.000	0.017	0.024
Province						
Newfoundland and Labrador	0.001	0.002	0.900	0.368	− 0.003	0.006
Nova Scotia	0.003	0.001	2.370	0.018	0.000	0.007
Quebec	0.002	0.001	1.720	0.086	− 0.001	0.005
Ontario (ref)						
Manitoba	0.003	0.001	2.010	0.044	− 0.001	0.006
Alberta	0.002	0.001	1.530	0.125	− 0.001	0.006
British Columbia	0.008	0.001	7.160	0.000	0.005	0.011
Rurality						
Urban (ref)						
Rural	− 0.001	0.001	− 0.420	0.676	− 0.004	0.003
Constant	0.507	0.089	5.680	0.000	0.277	0.737
N	29,038					
Adjusted R-squared	0.509					

Canadian Longitudinal Study on Aging Comprehensive, baseline data, Unweighted.
